# Annotation of Differentially Expressed Genes in the Somatic Embryogenesis of *Musa* and Their Location in the Banana Genome

**DOI:** 10.1155/2013/535737

**Published:** 2013-08-21

**Authors:** Josefina Ines Maldonado-Borges, José Roberto Ku-Cauich, Rosa Maria Escobedo-GraciaMedrano

**Affiliations:** Unidad de Bioquímica y Biología Molecular de Plantas, Centro de Investigación Científica de Yucatán, A.C., C 43 No. 130, Col. Chuburná de Hidalgo, Mérida, Yucatán, C.P. 97200, Mexico

## Abstract

Analysis of cDNA-AFLP was used to study the genes expressed in zygotic and somatic embryogenesis of *Musa acuminata* Colla ssp. *malaccensis*, and a comparison was made between their differential transcribed fragments (TDFs) and the sequenced genome of the double haploid- (DH-) Pahang of the *malaccensis* subspecies that is available in the network. A total of 253 transcript-derived fragments (TDFs) were detected with apparent size of 100–4000 bp using 5 pairs of AFLP primers, of which 21 were differentially expressed during the different stages of banana embryogenesis; 15 of the sequences have matched DH-Pahang chromosomes, with 7 of them being homologous to gene sequences encoding either known or putative protein domains of higher plants. Four TDF sequences were located in all *Musa* chromosomes, while the rest were located in one or two chromosomes. Their putative individual function is briefly reviewed based on published information, and the potential roles of these genes in embryo development are discussed. Thus the availability of the genome of *Musa* and the information of TDFs sequences presented here opens new possibilities for an in-depth study of the molecular and biochemical research of zygotic and somatic embryogenesis of *Musa*.

## 1. Introduction

Somatic embryogenesis is a powerful tool for the massive production of elite plant materials, as well as for molecular agricultural breeding through the use of biotechnological strategies. Although this technology can be applied to any plant species, it is particularly valuable for the asexually propagated ones, such as bananas (*Musa *spp.). Bananas are one of the most consumed fruits worldwide and represent an important source of revenue for tropical countries where they also account for one of the main staple foods. In spite of their nutritional and socioeconomic importance, molecular tools for genetic improvement of bananas are limited in comparison to other plant species. In addition, the molecular bases of zygotic and somatic embryogenesis in *Musa* are not fully understood. 

Recently, the genomic sequence of the double haploid banana-Pahang CIRAD 930 ITC 1511 (hereafter ITC 1511) was recently released [1]. ITC 1511 is derived from the Pahang wild diploid (2*n* = 22) *Musa acuminata* Colla. ssp. *malaccensis* accession which shares its genetic lineage with dessert and cooking bananas. The DH-Pahang genome size is 523 Mb (1C estimated through flow cytometry), and in a 91% assemblage it revealed 36,542 protein-coding genes anchored to the 11 *Musa* chromosomes. This provides a unique platform for genetic improvement of this underresearched vital crop. Besides the protein-coding genes, those for 235 microRNAs (*MIR*), corresponding to 37 different families, were found, including the eight families typical of Poaceae [1]. 

Using *M. acuminata *ssp. *malaccensis* immature zygotic embryos (IZE) we have developed an efficient somatic embryogenesis protocol, based on modifications to the one previously reported [2]. Moreover, we have observed changes in gene expression patterns during this process. Here, taking advantage of the information made available regarding the *Musa* genome, we were able to assign putative functions to some of these genes as well as to localize their position into the chromosomes of the *Musa* genome available. Our finding suggest that the cDNA-AFLP procedure was useful for identifying expressed genes during early and late zygotic and somatic embryogenesis in *M. acuminata *ssp*. malaccensis* and helping annotate them in the *Musa *genome, such as cytidine triphosphate synthase 2 (CTP synthase 2), serine/threonine protein kinase, starch branching enzyme (SBE1), early responsive to dehydration (ERD) and indole-3-acetic-acid-amido synthetase (GH3.1); additional work in this line is underway. 

## 2. Material and Methods

### 2.1. Plant Material and Establishment of Somatic Embryogenesis

In our study immature and mature zygotic embryos of *Musa acuminata *ssp*. malaccensis* (ITC 250) were obtained from fruits harvested from CICY's collection, located at the Experimental Station of the Instituto Nacional de Investigaciones Forestales (INIFAP) in Uxmal (Yucatán, Mexico; 20°21′34′′N 89°46′17′′W).

Embryogenic callus cultures were initiated from immature zygotic embryos (IZE) from fruits collected 60 to 65 days after-anthesis (DPA); embryos were extracted aseptically and cultured as described by Navarro et al. [2], except that for induction medium (MI) auxin was reduced to 4.5 mM of 2,4-dichlorophenoxyacetic acid (2,4-D). After three months in culture, cell suspensions were initiated from embryogenic calli in Cote liquid media M2 [3] and equal concentration of 2,4-D. Cultures were kept in the dark, in a shaker at 90 rpm, with media refreshed every two weeks. For embryo development, five-day-old cell suspensions were sieved through 60-mesh screen (230 *μ*m), and 250 *μ*L of the homogeneous embryogenic cell suspension (ECS), representing a 3% packed cell volume (PCV), was overlaid onto maturation media (MM) lacking plant growth regulator, either in agar or a disc of filter paper (Whatman no. 1, 9.0 cm in diameter) [2] in Petri dishes (100 × 15 mm) for a period of 45 to 60 days. Embryos were classified according to development as immature (globular in shape of whitish translucent appearance) and mature (torpedo-like, white opaque with a cotydelonary slit). Somatic mature embryos were germinated on Murashige and Skoog [4] media (MG) using 2.0 *μ*M 6-BA and 2.85 *μ*M IAA [2].

### 2.2. Collection of Zygotic and Somatic Embryogenesis Stages for RNA Extraction

 For this study immature (between 60 and 65 dpa) and mature (between 90 and 100 dpa) zygotic embryos were collected along with immature and mature somatic embryos. The different developmental stages were defined by histological [2] and scanning electron microscopy (SEM) assessment [5] (see Figure 2). Tissues at different developmental stages (immature zygotic (IZE), mature zygotic (MZE) embryos, embryogenic callus (EC), immature somatic (ISE), and mature somatic (MSE) embryos) were collected and rapidly freezed in liquid nitrogen, and samples were kept at −80°C until used for RNA extraction.

### 2.3. Differential Gene Expression

Gene expression at the different developmental stages of zygotic and somatic embryos (Figure 2) was analyzed by cDNA-AFLP. Total RNA from the different embryogenic phases was prepared using trizol reagent (Invitrogen, USA) and after some adjustments followed the protocol of Chomczynski and Saccchi (1987). RNA extracts were treated with RNase-free DNase I (Invitrogen), and first-strand cDNA was synthesized using Superscript II reverse transcriptase (Invitrogen, Carlsbad, CA, USA) using random primers. cDNA-AFLP was performed according to Vos et al. [6] and Bachem et al. [7], with modifications and using AFLP primers set (Table 1). The selective amplification primers were selected based on the high polymorphism previously shown for different banana species, including *M. acuminata *ssp*. malaccensis* [8]. cDNA was first digested with Mse I and then with Eco RI. Adaptors for both enzymes were then ligated to the extremes of the restriction fragments, in order to generate the substrates for amplification. Twenty rounds of preamplification were performed using AFLP primers with selective nucleotides (**C** for MseI and **A** for EcoRI, resp.). Reaction mixture (25 *μ*L) was prepared with 2.5 *μ*L of 10x PCR buffer, 0.75 *μ*L of 50 mM MgCl_2_, 0.75 *μ*L of each 30 *μ*M primer solution, 2.5 *μ*L of a 2 mM dNTPs, 5 *μ*L of the cDNA ligated and 1 : 10 diluted, and 0.125 *μ*L (5U) of Taq-DNA pol (Invitrogen). PCR cycles were at 92° (1 min), 56° (30 seg), and 72°C (1 min) for denaturalization, primer alignment, and amplification, respectively. Products were diluted (1 : 10), and 5 mL was amplified using five different primer combinations (Figure 2). These combinations already detected a high degree of polymorphism in *M. acuminata *ssp*. malaccensis* [8]. Reaction mixtures were prepared as described previously, but 0.38 *μ*L of 20 *μ*M primer solutions was added. PCR cycles (35) were at 94° (30 seg), 65° (30 seg), and 72°C (1 min) for denaturalization, primer alignment, and amplification, respectively. PCR products were mixed with an equal volume of loading buffer (0.01% bromophenol blue, 0.01% xylene cyanol, and 10 mM EDTA in 98% formaldehyde, pH 8.0), denatured at 95°C, and then kept in ice. Samples were electrophoresed in 6% polyacrylamide denaturing gels with TBE 1.0x (89 mM Tris pH 7.6, 89 mM boric acid, and 2 mM EDTA), at 55 W, 2000 V, 50 mA. Gels were stained with silver nitrate [9], dried, and digitalized for band analysis. The presence or absence of differential bands was registered for the different developmental stages and primer combinations (Figure 2). 

### 2.4. Transcript-Derived Fragment (TDF) Isolation and Reamplification

 The differentially expressed TDFs were assigned based on presence, absence, or differences of intensity and were cut with a sharp blade from the gel with care to avoid contaminations prior elution in 25 *μ*L of PCR buffer 2x. Aliquots of 2 *μ*L were reamplified as described previously using the same set of primers and PCR conditions as used for preamplification. Amplicons were resolved in 1.2% agarose gels; each single band was isolated and eluted using the QIAEX II Gel Extraction package (QUIAGEN). 

### 2.5. Cloning and Sequencing of TDFs

 Eluted TDFs were cloned into the plasmid pGEM-T Easy vector (Promega, Madison, WI, USA) and used to transform *E. coli* DH*α*5 cells. The cloned cDNA fragments were sequenced using a commercial service (Macrogen Inc., Seol, Republic of Korea). Sequences of TDF were cleaned by trimming off the plasmid sequences and then analyzed for homology against the NCBI database. TDFs were also compared to the *Musa* genome database (http://banana-genome.cirad.fr; [1]) to assign putative identities and function. 

## 3. Results

**Figure 1:**
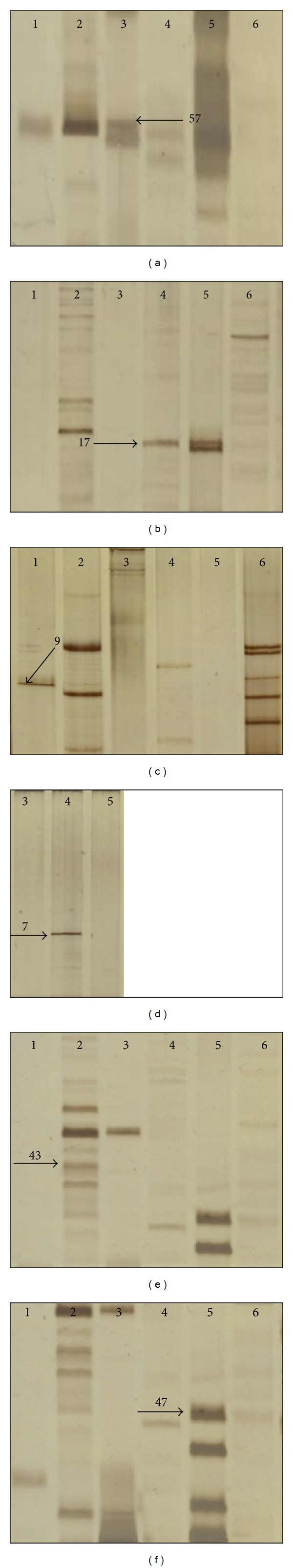
cDNA-AFLP patterns: zygotic versus somatic embryogenesis stages of *M. acuminata *ssp. *malaccensis*. Arrows with numbers showed TDF differentially expressed. (a) Early embryogenesis, (b) late embryogenesis, (c) immature zygotic embryo, (IZE), (d) mature zygotic embryo (MZE), (e) immature somatic embryo (ISE), and (f) matured somatic embryo (MSE). The numbers are 1: IZE, 2: ISE, 3: ECS, 4: MZE, 5: MSE, and 6: germinated somatic embryo (GSE).

**Figure 2:**
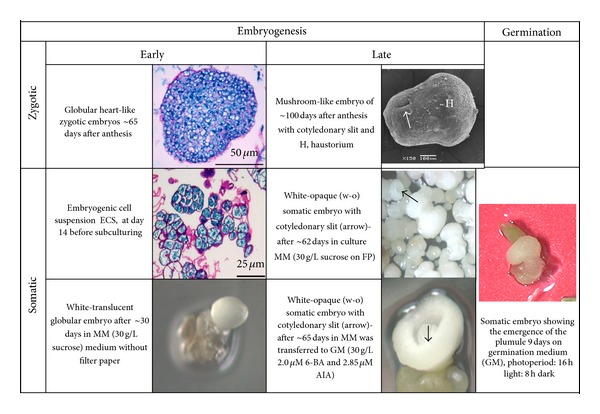
Different stages of zygotic and somatic embryogenesis of *M. acuminata *ssp. *malaccensis* plant material used for cDNA-AFLP analysis.

cDNA-AFLP analysis of the RNA samples from immature and mature zygotic and somatic embryos stages as well as embryogenic cell suspension culture materials of *M. acuminata *ssp*. malaccensis* (Figure 2) with five pairs of primers (Table 1) resulted in the identification of a total of 253 TDFs with a range in size from 100 to 4000 bp. Of these, 21 TDFs were clear and unambiguously differentially expressed through the process of banana embryogenesis (see Figures 1(a)–1(f), and Figure 2). These 21 TDFs differentially expressed ranged from 76 to 299 bp. Comparisons of the *Musa* DH-Pahang genome allowed to assign location of 15 of them on the *Musa* chromosomes, as well as possible identity (Table 2). Seven of these sequences corresponded to typical protein domains of higher plants and *Musa*. Interestingly, four sequences were located in all the *Musa *chromosomes, and two of them, namely, 17-1-5 and 47-1-4, were found only in mature embryos. The former of them occurred both in MSE (Figure 1(f), line 5) and MZE (Figure 1(b), lines 4 and 5), whereas the latter was only detected in MSE. The last two TDFs (24-2-1 and 46-5-4) occurred at all stages of the embryogenesis process, regardless of their origin, zygotic or somatic (Table 2). The remaining eleven sequences were located in either one or two chromosomes; six of these sequences were only found in ECS. 

## 4. Discussion

The understanding of the type and number of genes differentially expressed during embryogenesis in *Musa* would help discerning the molecular mechanisms involved during the passage through the different stages involved in the processes, both of zygotic and somatic origins. It also opens the path towards biotechnological fundamental studies. To our knowledge this work represents one of the first steps in that direction. The results shown here contribute to the allocation of the putative function and participation of genes identified during the sequencing of the DH-Pahang *Musa* genome [1]. 

In this study, somatic embryogenesis induced *in vitro* and zygotic embryogenesis from collected stages of fruits with seeds of *M. acuminata *ssp. *malaccensis* plants allowed the identification of genes expressed during both important processes. Important differences in TDFs were observed among stages of zygotic and somatic embryogenesis of *M. acuminata *ssp.* malaccensis*. TDFs differential patterns corresponded to genes involved both in primary and secondary metabolisms, signal transduction, gene regulation, energy metabolism, and defense and cellular processes. Out of the 253 differential TDFs, only 15 could be located on the chromosomes and showed between 88 and 100% identity to available *Musa* genome sequences, thereby suggesting that most of the TDFs in the current study represent genes of banana related to embryogenesis. 

Interestingly, TDFs of cytidine triphosphate synthase 2 (CTPS-2) were found in mature zygotic embryo as well as in proembryo and embryogenic cells suspensions. This protein is involved in the metabolism of pyrimidine and it has not been studied in depth within plants, although five gene copies encoding this protein have been identified in *Arabidopsis* [10]; two cDNAs were found to be upregulated during the ripening of apples [11]. Pyrimidine, like purine nucleotides, represents fundamental compounds, central to both primary and secondary plant metabolisms. Since it is involved in different cellular processes, pyrimidine is considered of vital importance for plant growth, development, and reproduction, during germination, pollen tube growth, flowering, and seed formation. Moreover, carbohydrate metabolism is closely linked to pyrimidine nucleotides since many enzymes involved in carbohydrate interconvention require this nucleotide as a substrate. Besides the synthesis of important cellular metabolites such as cell wall polysaccharides and glycoproteins, glycolipids and sulfolipids require pyrimidine nucleotides for their production; therefore a regulatory link between the levels of pyrimidine nucleotides and a large number of cellular biochemical processes require to be further explored [10]. 

TDFs representing genes for protein serine/threonine kinase involved in signal transduction were found in proembryos and embryogenic cells from suspension cultures. Protein phosphorylation, catalyzed by protein kinases, is one of the most fundamental regulatory mechanisms known to control protein activity and cellular signaling [12]. The network of these proteins in the plant cell appears to act as a “central processing unit” which accepts the receptors' information, recognizes changes in environmental conditions, that is, plant growth regulators, and other external factors, and converts the information into a suitable signal such as changes in metabolism, gene expression, and cell growth and division [13].

 In addition, TDFs with differential expression for starch branching enzyme (1,4-*α*-glucan-branching enzyme 2, chloroplastic/amyloplastic ~(SBE1)), an enzyme that participates in starch metabolism, indicate its involvement in all stages of somatic embryogenesis in *Musa*. Starch is the major carbohydrate reserve of plant cells. The synthesis of amylopectin, one of the two major components of starch, is controlled by the activity of enzymes of three components: the starch synthase, starch branching, and starch disbranching enzymes [14]. Starch branching enzyme (SBE) plays an important role in starch biosynthesis by introducing branch points, the *α*-1,6 linkages in starch. Studies in maize indicate that different isoforms of *Sbe* are independently controlled; that is, *Sbe2b* appears to be endosperm specific, whereas *Sbe2a* form is at high levels in embryo than endosperm, and the absence of *(SBE1)* is associated with altered physiological function of starch [15]. In our case, we did not detect TDFs for starch branching enzyme during stages of zygotic embryogenesis, though it is possible as in maize that similar events could be related to the different zygotic embryo stages, a fact that merits further study, and the role that this enzyme(s) is taking during somatic embryo development. Also during somatic embryogenesis TDFs for the signal peptide peptidase-3 were detected; the signal peptide peptidases (SPP) are members of a family of proteases that are responsible for intramembrane processing of other proteins during the intracellular signaling events. In *Arabidopsis thaliana* six genes encoding these proteins were found; their physiological functions are not fully known [16], but it seems to require for male gametophyte development maturation of the pollen and its germination. In *Musa*, the physiological role of the signal peptide peptidase and their substrates continues to be unknown. 

 TDFs related to early-responsive to dehydration (ERD) proteins were found in mature somatic embryos and embryogenic cell suspension (MZE and ECS) cultures of *M. acuminata *ssp. *malaccensis*. The *ERD* genes are defined as genes rapidly activated during drought stress. The encoded proteins show great structural and functional diversity and are the first line of defense against drought stress in plants [17]. To date, a total of 16 complementary DNA (cDNA) for *ERD* genes have been isolated from *Arabidopsis thaliana,* and only half of these have been characterized in soybean. Such genes encode proteins that include ClpA/B ATP-dependent protease, heat shock proteins HSP-70-1, methionine-dependent methyltransferases, membrane proteins, proline dehydrogenase, carbohydrate transporters, senescence-related genes, glutathione-S transferase type LEA proteins (Late Embryogenesis Abundant), jasmonic acid biosynthesis proteins, chloroplast proteins, and hydrophilic and extension ubiquitin proteins. Regarding the expression controlled by phytohormones ERD genes have several functions in response to ABA signaling during germination and development and/or are involved in stress tolerance. Some genes may be induced in response to more than one phytohormone. The common characteristic of these genes is that their expression is increased rapidly in response to environmental stress; it is also suggested that such genes may function to regulate the expression of effectors' proteins and signaling pathways in response to stress [18]. In our case the presence of TDFs related to ERD was consistent with MSE; during SE maturation there was a restriction of water availability owing to the use of a filter paper between the developing embryos and the culture medium, perhaps with an increase in the hormone ABA due to drought stress imposed prior to germination, while in embryogenic cell suspension cultures probably the osmotic pressure by sucrose in the medium and/or the concentration of the added exogenous auxin act as abiotic stress that result in the expression of the TDFs related to ERDs.

In ECS of* M. acuminata *ssp.* malaccensis *we found TDFs recognized as BSD-domain containing proteins, which belong to a family of transcription factors, TFs. The BSD domain is characterized by three *α* helices, probably involved in DNA binding, and by conserved tryptophan and phenylalanine residues located at the C-terminus of the domain [19]. The BSD domain is associated with basal transcription factors, proteins linked with synapses, and different hypothetical proteins present in a variety of species ranging from protozoans to humans [20]. Thus it is likely that the BSD-domain containing protein found here represents basal TFs associated with cell proliferation during somatic embryogenesis *Musa *as well as other plant species. 

In this study TDFs differentially expressed in ECS of *Musa* were found to be related to the indole-3-acetic acid-amido synthetase (GH3.1) (Aux/IAA, amido synthetase, GH3) gene, such protein is also called auxin-responsive GH3-like protein, it is involved in the catalysis of the synthesis of IAA-amino acid conjugates, providing a mechanism for the plant to cope with the presence of excess auxin. Maintaining homeostasis through converting free IAA to IAA conjugated with carbohydrates, amino and methyl groups forms are a conserved mechanism in monocots and dicots. The GH3 family proteins are responsible for converting the active IAA to its inactive form by conjugating the amino acid free IAA. The members of this gene family in *Arabidopsis* are regulated by hormones and environmental factors, including salicylic acid, abscisic acid, and light pathogen infection [21]. Auxin regulates the growth and development of plants by altering the expression of various genes, including genes such as GH3 widely studied in dicots, but little information is available in monocots. In rice 12 members of GH3 family genes have been identified; transcripts abundance increased by auxin treatments, sustaining a role in signal transduction pathway [22]. In cell suspension cultures where we assume that this enzyme was produced by the presence of auxin during culture and has been associated with this signaling pathway, further work will elucidate their role in banana embryogenesis. 

In conclusion, the procedure of cDNA-AFLP was useful for identifying genes expressed during early and late zygotic and somatic embryogenesis in *M. acuminata *ssp.* malaccensis*, compared the events, and annotated their location in the available DH-Pahang sequence genome. This represents a contribution to the known genetic changes that lie behind this process in monocotyledons since the allocation of genes not currently recognized as involved in such biological processes or metabolic pathways is suggested. Additionally, we have identified a number of TDFs with significantly lower expression levels in ESC and IZE; these could encode an interesting candidate proteins involved in embryogenesis.

## Figures and Tables

**Table 1:** Set of AFLP primer and adaptor pairs used for the ligation: preselective and selective amplification of cDNA (in bold: selective nucleotides, PA: preselective primers for amplification).

Primer pair	Adapter/primer	Sequence 5′-3′	
	Adp.1 eEcoR1	CTCGTAGACTGCGTACC	
	Adp.2 eEcoR1	AATTGGTACGCAGTCT	
	Adp.1 eMse I	GACGATGAGTCCTGAG	
	Adp.2 eMse I	TACTCAGGACTCAT	
	PA-eEcoRI-01	GACTGCGTACCAATTCC	
	PA-eMse I-02	GATGAGTCCTGAGTAAC	

		**EcoR1, 3+**	**Mse I, 3+**

(1) E4-M1		GACTGCGTACCAATTC**ACG**	GATGAGTCCTGATAA**CAA**
(2) E1-M3		GACTGCGTACCAATTC**AAC**	GATGAGTCCTGATAA**CAG**
(3) E4C-M10		GACTGCGTACCAATTC**CAC**	GATGAGTCCTGATAA**CCG**
(4) E7C-M10		GACTGCGTACCAATTC**CGC**	GATGAGTCCTGATAA**CCG**
(5) E15C-M10		GACTGCGTACCAATTC**CTC**	GATGAGTCCTGATAA**CCG**

**Table 2:** Annotation of differentially expressed genes during different stages of somatic versus zygotic embryogenesis in *Musa acuminata* ssp. *malaccensis* and their location in the chromosomes of banana genome.

TDF number (base pairs)	Hit to the *Musa *chromosome no.	*E* value	% identity	Protein	Embryogenesis stage
7-1-2 (88 )	6	9*E* − 21	95.24	CTP synthase 2	MZE
7-1-4 (71)	5	4*E* − 13	97.62	Serine/threonine-protein kinase	ECS
17-1-5 (299)	All	2*E* − 55	99.12	—	MSE, MZE
24-2-1 (286)	6	5*E* − 56	99.0	1,4-*α*-glucan-branching enzyme 2 chloroplastic/amyloplastic ~SBE1	All stages of somatic embryogenesis
35-2-3 (225)	1	1*E* − 107	99.5	—	IZE
43-1-1 (163)	10	8*E* − 66	99.24	—	IZE
46-5-4 (119)	All	2*E* − 22	89.36	Putative signal peptide peptidase-like 3	All stages of somatic embryogenesis
47-1-2 (148)	7	3*E* − 59	100	—	MSE
47-1-4 (149)	All (6)	3*E* − 25	87.83	—	MSE
47-1-5 (192)	4	8*E* − 11	92.16	Early-responsive to dehydration protein-related	MSE
58-1-2 (94)	4	6*E* − 16	96.08	Early-responsive to dehydration protein-related,	ECS
58-1-3 (88)	6	9*E* − 21	95.24	CTP synthase 2	ECS
60-1-3 (71)	5	4*E* − 13	97.62	Serine/threonine-protein kinase	ECS
60-5-4 (120)	6	3*E* − 18	90.67	Putative BSD domain containing protein	ECS
60-5-7 (116)	7	2*E* − 44	100	Probably the indole-3-acetic-acid-amido synthetase GH3.1	ECS

MZE and MSE: matured zygotic and somatic embryo, IZE: immature zygotic embryo; ECS: embryogenic cell suspension.
